# Supplementary material for: Experimental evidence for cancer resistance in a bat species
Source: Nat Commun. 2024 Feb 15;15:1401. doi: 10.1038/s41467-024-45767-1 (PMC10869793; doi:10.1038/s41467-024-45767-1)
Supplement: Supplementary file 1 — Supplementary Information [file 41467_2024_45767_MOESM1_ESM.pdf]

Supplementary Information

Experimental evidence for cancer resistance in a bat species

Rong Hua[1,2,4], Yuan-Shuo Ma[1,2,4], Lu Yang[1,2,4], Jun-Jun Hao[1], Qin-Yang Hua[1,2], Lu-Ye Shi[1], Xiao-Qing Yao[1,2], Hao-Yu Zhi[1,2] & Zhen Liu[1,3*]

[1]State Key Laboratory of Genetic Resources and Evolution, Kunming Institute of Zoology, Chinese Academy of Sciences, Kunming, China

[2]Kunming College of Life Science, University of Chinese Academy of Sciences, Beijing, China

[3]Yunnan Key Laboratory of Biodiversity Information, Kunming, China

[4]These authors contributed equally: Rong Hua, Yuan-Shuo Ma, & Lu Yang

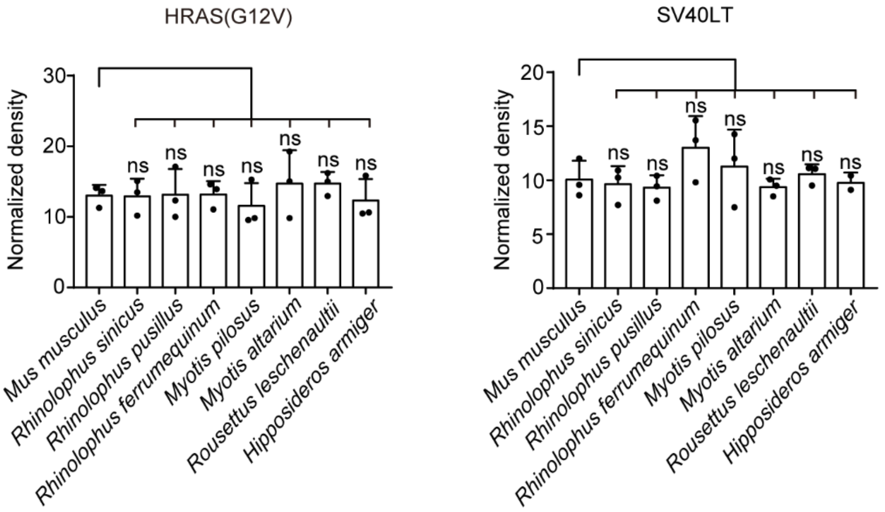

Supplementary Fig. 1. The comparisons of the protein levels of HRAS(G12V) and SV40 LT stably expressing in fibroblasts across seven bat species as well as mouse. β-actin was used as a control. The experiment was repeated independently at least three times with similar results. The *P* values are from two-tailed Student's *t*-tests. ns, not significant.

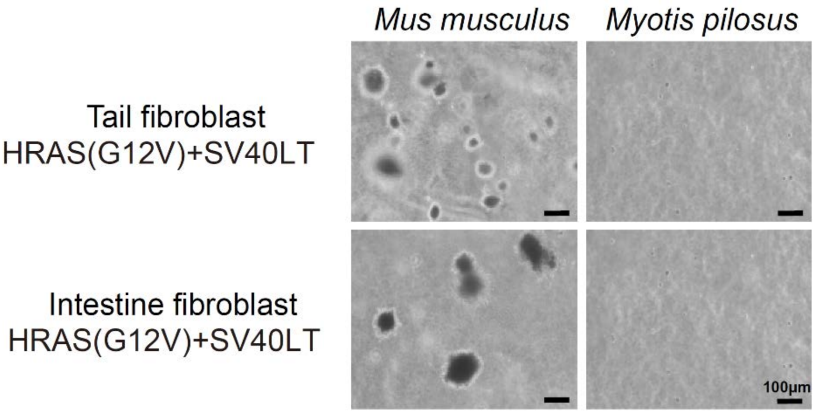

Supplementary Fig. 2. Anchorage-independent growth assay of the tail and intestine fibroblasts expressing HRAS(G12V) and SV40 LT for MPI and mouse. Compared with mouse, the fibroblasts from different MPI tissues consistently form remarkably small colonies. The representative microphotographs of colonies grown in soft agar after 4 weeks at 10× magnification are shown. Scale bar: 100μm.

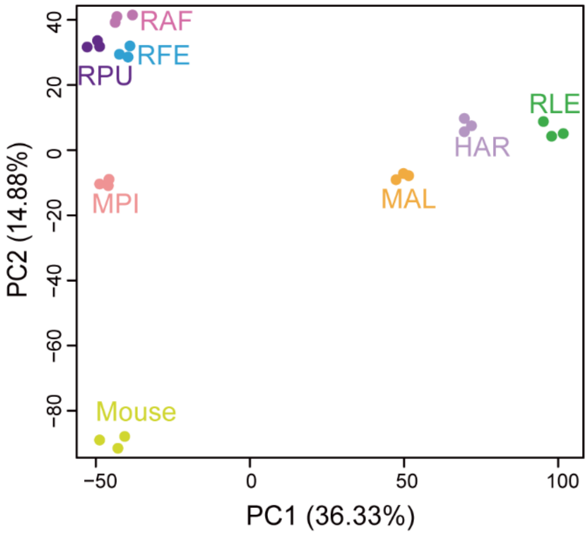

Supplementary Fig. 3. Principal component analysis of RNA-seq data across 24 fibroblast samples from eight different species. Three biological replicates from the same species tended to cluster together. Different colors represented different species.

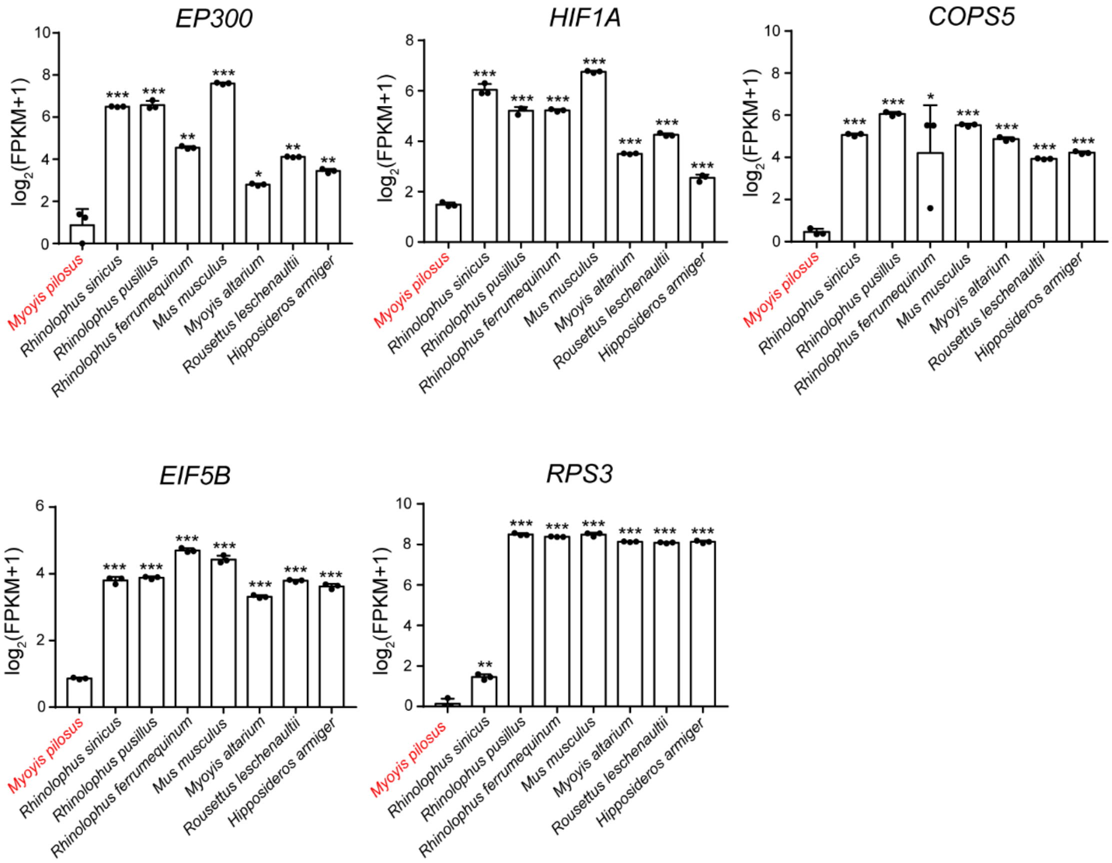

Supplementary Fig. 4. The top 5 genes, including *EP300*, *HIF1A*, *COPS5*, *EIF5B*, and *RPS3*, are significantly downregulated in MPI compared to mouse and other bat species. The number of dots ($n = 3$) represents the number of biologically independent samples. The P values are from two-tailed Student's t-tests. *$P < 0.05$, **$P < 0.01$, ***$P < 0.001$.

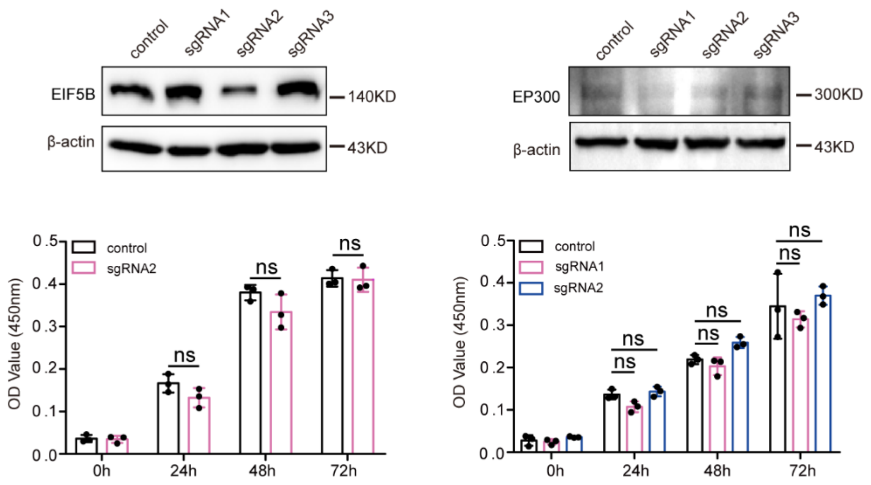

Supplementary Fig. 5. Immunoblotting shows the downregulation of the protein levels of EP300 and EIF5B. The experiment was repeated independently at least three times with similar results. The downregulation of EP300 and EIF5B causes no remarkable differences in cell proliferation. The number of dots (n = 3) represents the number of biologically independent experiments. The P values are from two-tailed Student's t-tests. ns, not significant.

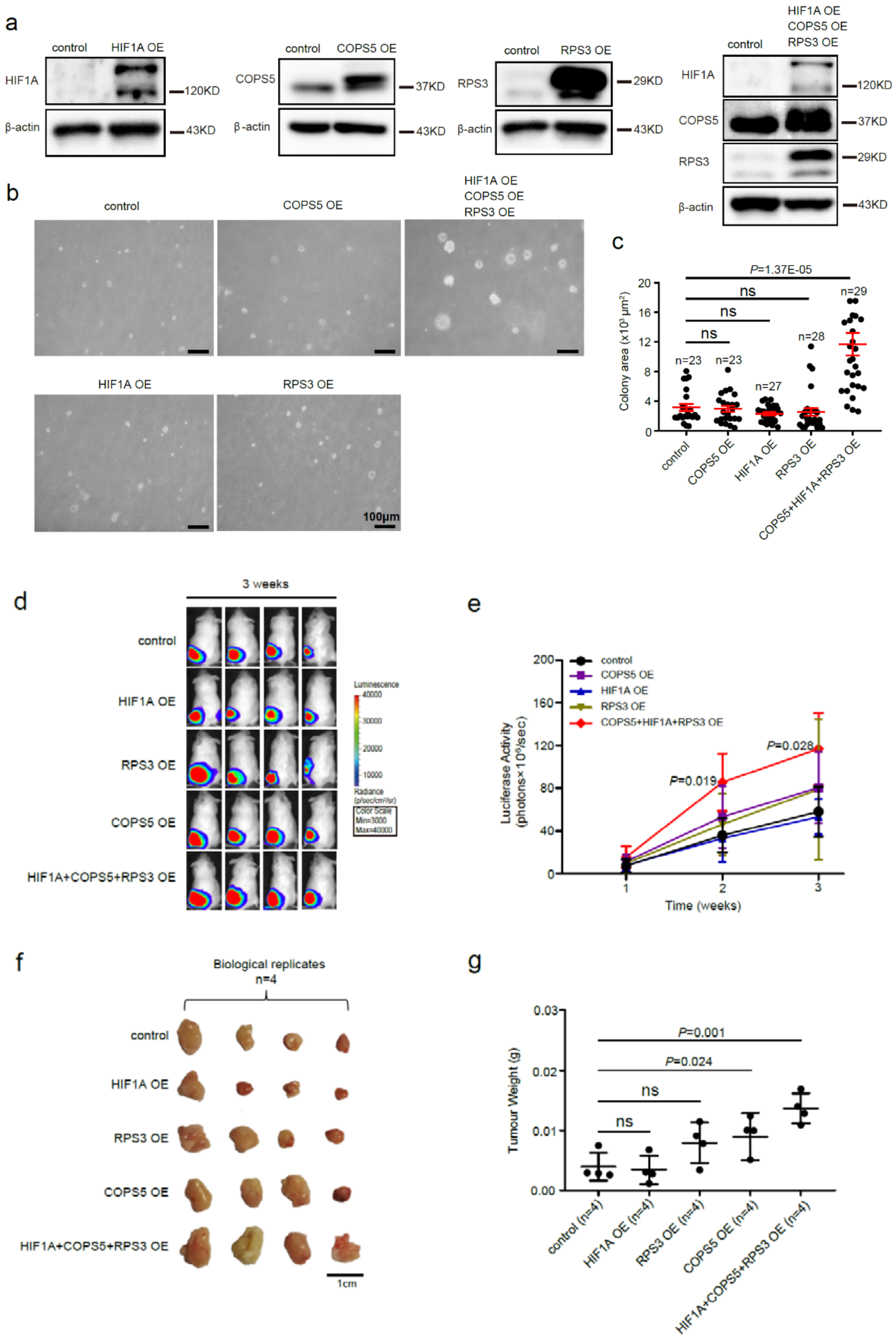
a
control
HIF1A OE
HIF1A
β-actin
120KD
43KD
control
COPS5 OE
COPS5
β-actin
37KD
43KD
control
RPS3 OE
RPS3
β-actin
29KD
43KD
control
HIF1A OE
COPS5 OE
RPS3 OE
HIF1A
COPS5
RPS3
β-actin
120KD
37KD
29KD
43KD
b
control
COPS5 OE
HIF1A OE
COPS5 OE
RPS3 OE
HIF1A OE
RPS3 OE
100μm
c
P=1.37E-05
ns
ns
ns
n=23
n=23
n=27
n=28
n=29
Colony area (x10³ μm²)
0
4
8
12
16
20
control
COPS5 OE
HIF1A OE
RPS3 OE
COPS5+HIF1A+RPS3 OE
d
3 weeks
control
HIF1A OE
RPS3 OE
COPS5 OE
HIF1A+COPS5+RPS3 OE
Luminescence
40000
30000
20000
10000
Radiance
(p/sec/cm²/sr)
Color Scale
Min=3000
Max=40000
e
control
COPS5 OE
HIF1A OE
RPS3 OE
COPS5+HIF1A+RPS3 OE
Luciferase Activity
(photons×10⁶/sec)
0
40
80
120
160
200
P=0.019
P=0.028
1
2
3
Time (weeks)
f
Biological replicates
n=4
control
HIF1A OE
RPS3 OE
COPS5 OE
HIF1A+COPS5+RPS3 OE
1cm
g
P=0.001
P=0.024
ns
ns
Tumour Weight (g)
0.00
0.01
0.02
0.03
control (n=4)
HIF1A OE (n=4)
RPS3 OE (n=4)
COPS5 OE (n=4)
HIF1A+COPS5+RPS3 OE (n=4)

Supplementary Fig. 6. The overexpression of HIF1A, RPS3, and COPS5 enlarges tumor size. (**a**) Immunoblotting shows the overexpression of HIF1A, COPS5, and RPS3 in MPI-SF. OE means overexpression. The experiment was repeated independently at least three times with similar results. (**b**) The representative microphotographs of colonies from anchorage-independent growth assay for the MPI-SF overexpressing *HIF1A*, *COPS5*, and *RPS3*. (**c**) The combined overexpression of the three genes results in significantly larger soft agar colonies compared to other groups. The *n* values represent the numbers of colonies from three independent experiments. (**d**) Representative images of bioluminescence signals in immunodeficient mice bearing the xenografts derived from the MPI-SF overexpressing *HIF1A*, *COPS5*, and *RPS3* three weeks post subcutaneous implantation. (**e**) The luciferase activity is stronger for the xenografts derived from the MPI-SF combinedly overexpressing the three genes three weeks post subcutaneous implantation. (**f**) Representative images of the xenografts respectively derived from MPI-SF overexpressing the three genes three weeks post subcutaneous implantation. Scale bar: 1cm. (**g**) The xenografts derived from the MPI-SF combinedly overexpressing the three genes are heavier than those derived from the MPI-SF overexpressing individual genes three weeks post subcutaneous implantation. The number of dots represents the number of biologically independent animals. All data are presented as mean ± SD. The *P* values are from two-tailed Student's *t*-tests.

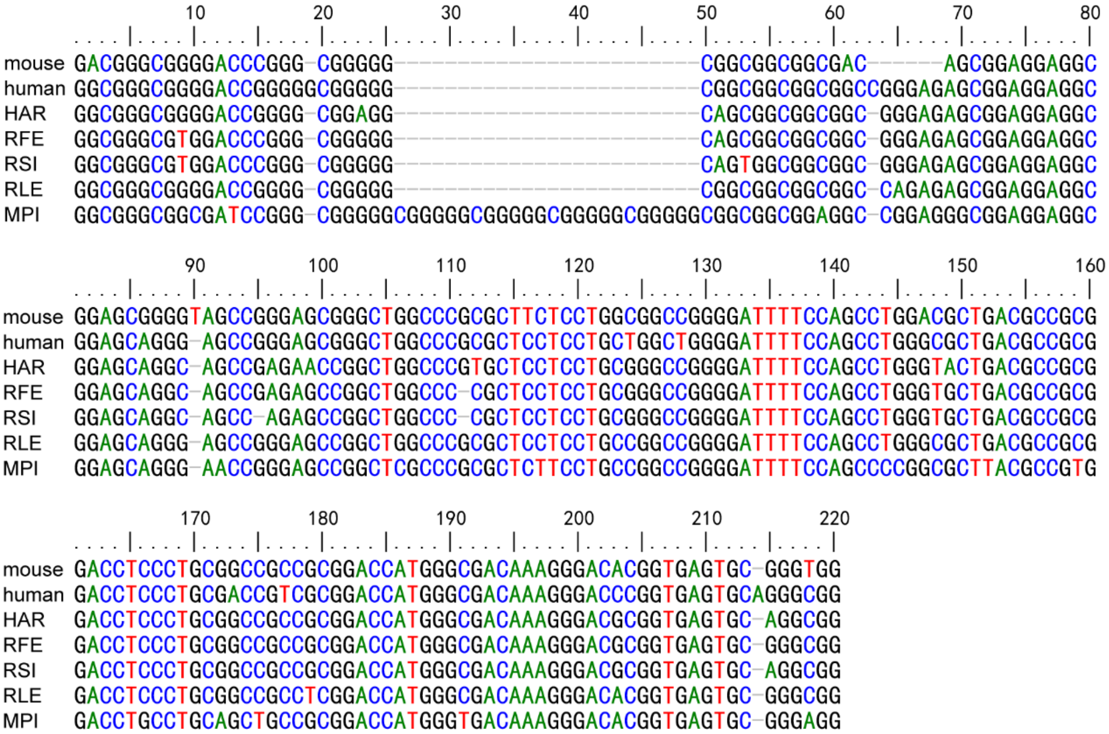

Supplementary Fig. 7. Sequence alignment of CNE563305 across eight species. HAR, the great leaf-nosed bat (*Hipposideros armiger*); RFE, the greater horseshoe bat (*Rhinolophus ferrumequinum*); RSI, the Chinese rufous horseshoe bat (*Rhinolophus sinicus*); RLE the Leschenault's Rousette (*Rousettus leschenaultii*); MPI, the big-footed bat (*Myotis pilosus*).

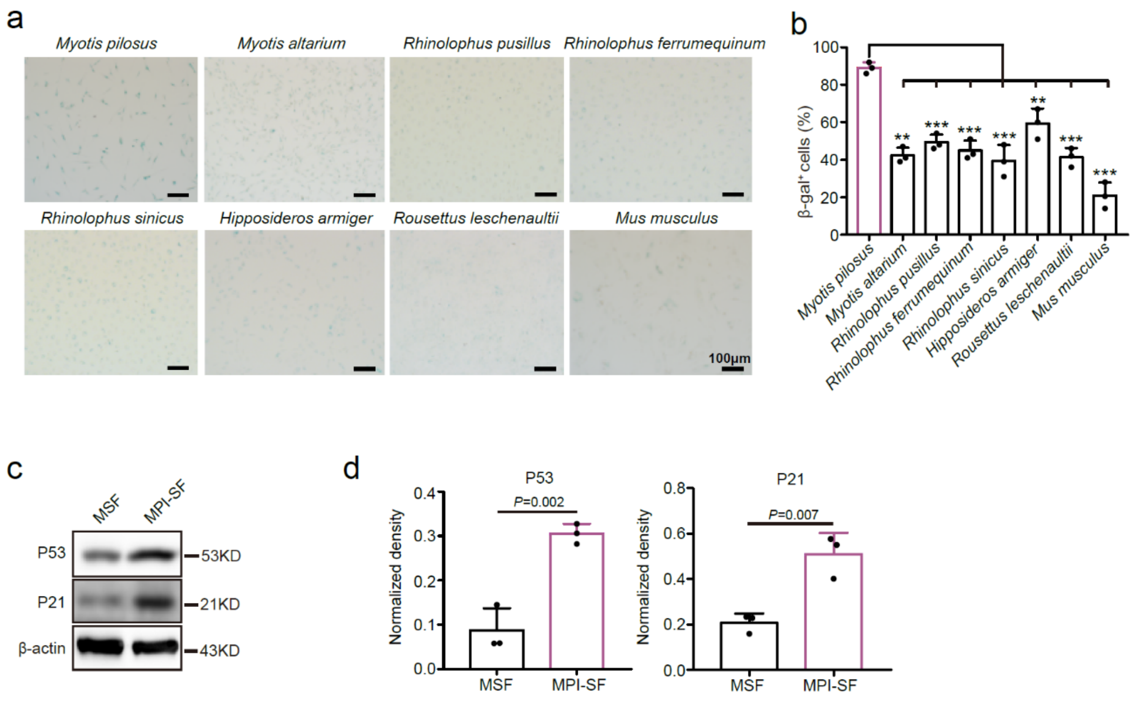

Supplementary Fig. 8. The experiments for cellular senescence. (**a**) Representative images of the fibroblasts derived from the eight species, which were treated with 10 μmol/L etoposide for 12 hours. (**b**) The proportion of SA-β-gal-positive fibroblasts across eight species. (**c**) Immunoblotting of P53 and P21 in MSF and MPI-SF. β-actin was used as a control. (**d**) The protein levels of P53 and P21 are significantly higher in MPI-SF than in MSF. The number of dots ($n = 3$) in (**b**) and (**d**) represents the number of biologically independent experiments. The P values are from two-tailed Student's t-tests. $^{**}P < 0.01$, $^{***}P < 0.001$.

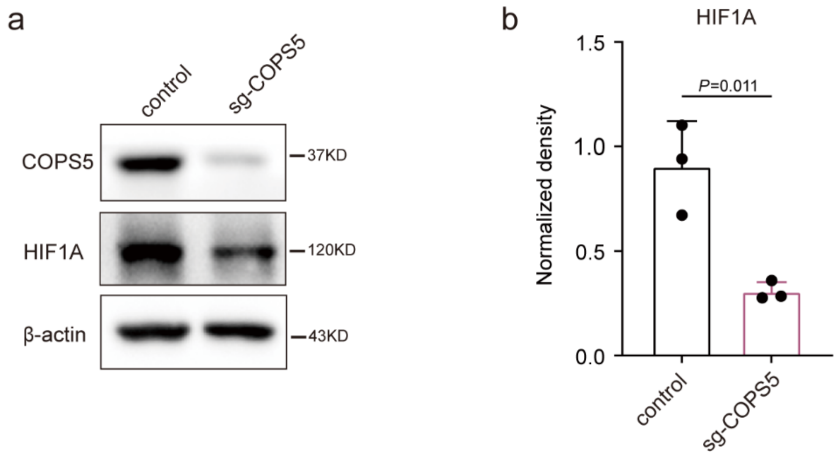

Supplementary Fig. 9. When the expression of *COPS5* is inhibited (**a**), HIF1A expression significantly decreases (**b**). The number of dots (n = 3) represents the number of biologically independent experiments. The P value is from the two-tailed Student's t-test.

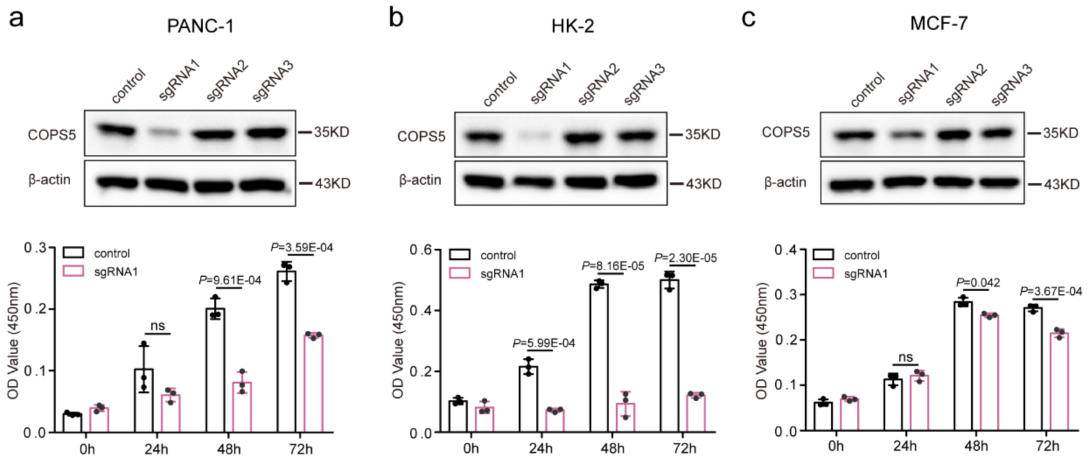

Supplementary Fig. 10. The expression of COPS5 is inhibited in a pancreatic cancer cell line derived from the pancreatic duct epithelium (PANC-1, **a**), the human proximal tubular epithelial cells (HK-2, **b**), and a breast cancer cell line derived from the mammary epithelium (MCF-7, **c**), respectively. These epithelial and tumor cell lines exhibit a significant decrease in proliferation. The number of dots (n = 3) represents the number of biologically independent experiments. The P values are from the two-tailed Student's t-tests.

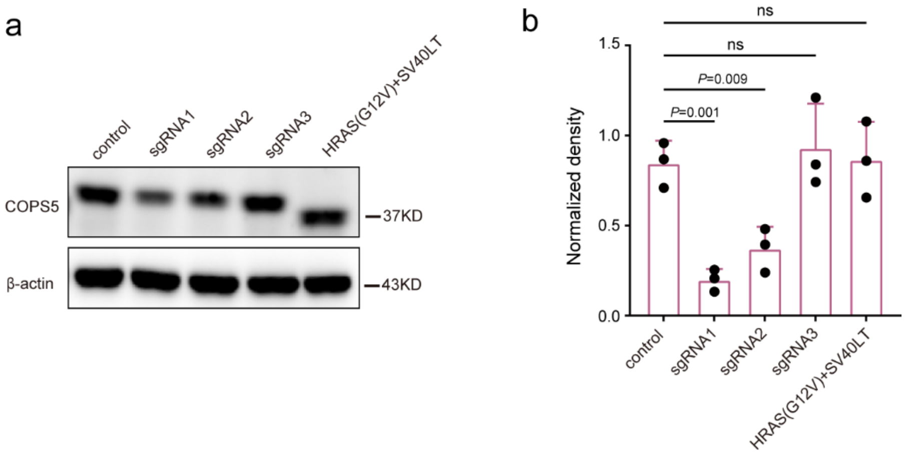

Supplementary Fig. 11. The inhibition of the expression of *COPS5* in MPI cells. (**a**) The expression of *COPS5* is inhibited by sgRNA1 and sgRNA2 in MPI cells. (**b**) The band intensity of COPS5 is significantly weakened for sgRNA1 and sgRNA2. The number of dots (n = 3) represents the number of biologically independent experiments. The P values are from the two-tailed Student's t-tests. ns, not significant

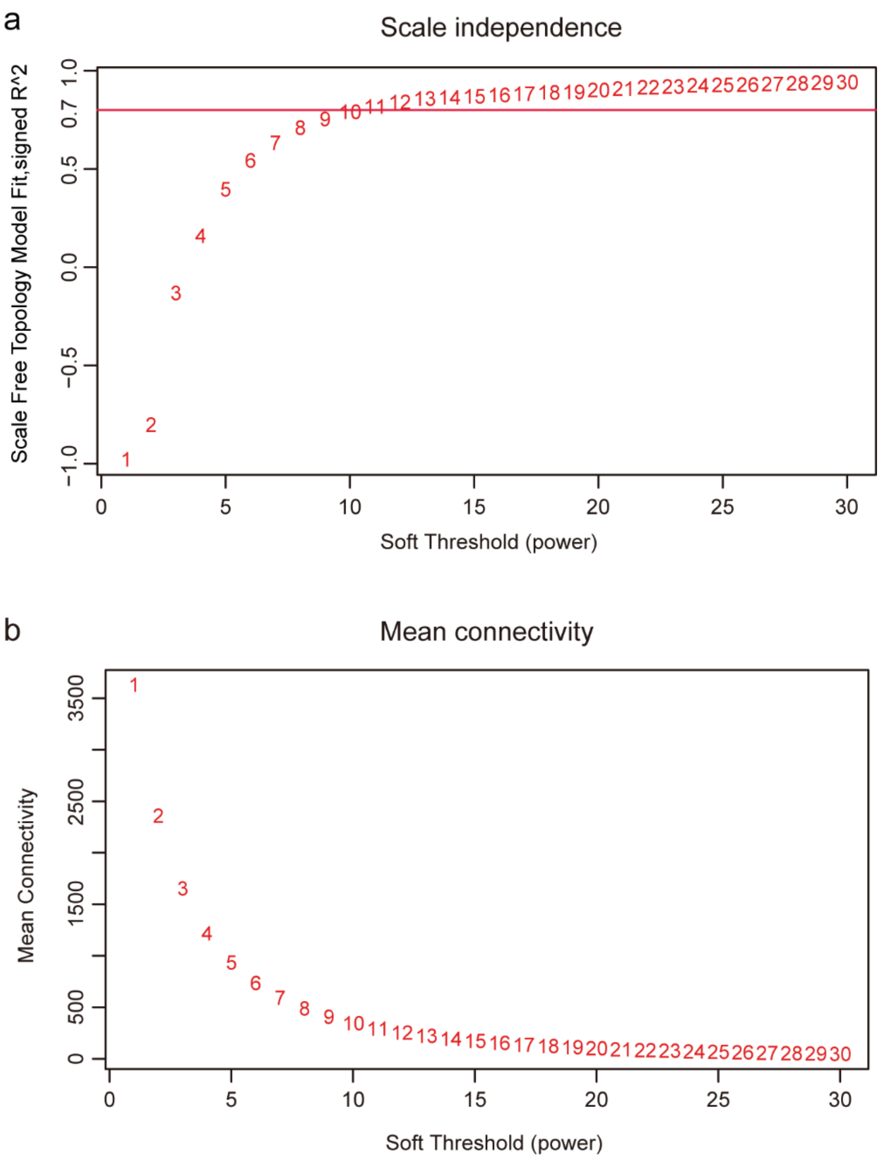

Supplementary Fig. 12. The index of soft threshold for WGCNA. (**a**) The scale-free fit index as a function of the soft-thresholding power. (**b**) The mean connectivity as a function of the soft-thresholding power.
