# Supplementary material for: Experimental evidence for cancer resistance in a bat species
Source: Nat Commun. 2024 Feb 15;15:1401. doi: 10.1038/s41467-024-45767-1 (PMC10869793; doi:10.1038/s41467-024-45767-1)
Supplement: Supplementary file 3 — Description of Additional Supplementary Files [file 41467_2024_45767_MOESM3_ESM.pdf]

<u>Description of Additional Supplementary Files</u>

Supplementary Data 1. A total of 34 gene co-expression modules identified with WGCNA.

Supplementary Data 2. Protein-protein interaction network based on the STRING database for the genes involved in M1 and M2.

Supplementary Data 3. Survival rates for 350 genes in M1 and M2 across 21 tumour types collected from the KMplot database.

Supplementary Data 4. Detection of positive selection along the branch of MPI.

Supplementary Data 5. Summary of the genome assembly of *Myotis pilosus*.

Supplementary Data 6. The genomic completeness of the *Myotis pilosus* genome with the BUSCO.

Supplementary Data 7. The 46 MSF-specific gained accessible peaks overlapping with ENCODE cCREs located within 300kb around *HIF1A*, *COPS5*, and *RPS3*.

Supplementary Data 8. Different types of transposons in the genome of MPI.

Supplementary Data 9. The sgRNA sequences for five genes.

Supplementary Data 10. The genome versions of 9 mammals used for homology-based gene prediction of MPI genome.

Supplementary Data 11. The genome versions of 6 mammals for genome alignment.

Supplementary Data 12. sgRNA sequences targeting mouse CNE143336.

Supplementary Data 13. The qPCR primers targeting the RSC sequences of mouse and MPI.
