# Supplementary material for: Experimental evidence for cancer resistance in a bat species
Source: Nat Commun. 2024 Feb 15;15:1401. doi: 10.1038/s41467-024-45767-1 (PMC10869793; doi:10.1038/s41467-024-45767-1)
Supplement: Supplementary file 17 — Reporting Summary [file 41467_2024_45767_MOESM17_ESM.pdf]

nature portfolio

Corresponding author(s): Zhen Liu

Last updated by author(s): Jan 20, 2024

Reporting Summary

Nature Portfolio wishes to improve the reproducibility of the work that we publish. This form provides structure for consistency and transparency in reporting. For further information on Nature Portfolio policies, see our Editorial Policies and the Editorial Policy Checklist.

Statistics

For all statistical analyses, confirm that the following items are present in the figure legend, table legend, main text, or Methods section.

n/a	Confirmed	
☐	☒	The exact sample size (*n*) for each experimental group/condition, given as a discrete number and unit of measurement
☐	☒	A statement on whether measurements were taken from distinct samples or whether the same sample was measured repeatedly
☐	☒	The statistical test(s) used AND whether they are one- or two-sided<br>*Only common tests should be described solely by name; describe more complex techniques in the Methods section.*
☐	☒	A description of all covariates tested
☐	☒	A description of any assumptions or corrections, such as tests of normality and adjustment for multiple comparisons
☐	☒	A full description of the statistical parameters including central tendency (e.g. means) or other basic estimates (e.g. regression coefficient) AND variation (e.g. standard deviation) or associated estimates of uncertainty (e.g. confidence intervals)
☐	☒	For null hypothesis testing, the test statistic (e.g. *F*, *t*, *r*) with confidence intervals, effect sizes, degrees of freedom and *P* value noted<br>*Give P values as exact values whenever suitable.*
☒	☐	For Bayesian analysis, information on the choice of priors and Markov chain Monte Carlo settings
☒	☐	For hierarchical and complex designs, identification of the appropriate level for tests and full reporting of outcomes
☐	☒	Estimates of effect sizes (e.g. Cohen's *d*, Pearson's *r*), indicating how they were calculated

Our web collection on statistics for biologists contains articles on many of the points above.

Software and code

Policy information about availability of computer code

Data collection	The immunoblotting images were obtained using the Tanon 5200 imaging system. The images of colonies and SA-β-gal cells were captured using the Nikon Ti-E inverted Microscope system. The quantification of immunoblotting and colonies area was performed using mage-pro plus 6.0 software. The bioluminescent imaging of xenografts was conducted using the IVIS system and the resulting images were analyzed using the Living Image software (Caliper Life Science, IVIS Lumina Xr). The absorbance value of cells was measured at 450 nm using a Hybrid reader (Synergy H1).
Data analysis	All experimental data were analyzed using GraphPad Prism7 and Excel.<br>The tools and packages : Trinity (version 2.8.5); signed Weighted Gene Co-expression Network Analysis (WGCNA, version 1.70); Cytoscape (version 3.7.2); NextDenovo (version 2.5.2); BUSCO (version 5.4.0); RepeatModeler (version 1.0.4); RepeatMasker (version 4.0.6); PASA (version 2.5.0); TblastN (version 2.2.29+); GeMoMa (version 1.6.1); EVM (version 2.1.0); Lastz (version 1.04.22); axtChain (version 3.0.2); PRANK (version 170427); PAML (version 4.7); PHAST (version 8.71); ENCODE pipeline (github.com/ENCODE-DCC/atac-seq-pipeline)(version 1.8.0); Bowtie2 (version2.5.0); Samtools (version 1.9); Picard (broadinstitute.github.io/picard)(version 2.20.7); BEDTools (version 2.30.0); MACS2 (version 2.2.4); haliftOver (version 2.2); Integrative Genomics Vviewer (version 2.8.7); wigToBigWi (version 357); Homer (version 4.11); FIMO (version 5.5.2). Our custom code is available on Zenodo at doi.org/10.5281/zenodo.10262324.

For manuscripts utilizing custom algorithms or software that are central to the research but not yet described in published literature, software must be made available to editors and reviewers. We strongly encourage code deposition in a community repository (e.g. GitHub). See the Nature Portfolio guidelines for submitting code & software for further information.

Data

Policy information about availability of data

All manuscripts must include a data availability statement. This statement should provide the following information, where applicable:

- Accession codes, unique identifiers, or web links for publicly available datasets
- A description of any restrictions on data availability
- For clinical datasets or third party data, please ensure that the statement adheres to our policy

The genomic and transcriptomic data generated have been deposited both in the NCBI database under accession codes: BioProject PRJNA976519, PRJNA975353, and PRJNA975438 [https://www.ncbi.nlm.nih.gov/bioproject/PRJNA976519/], and in the Science Data Bank [https://doi.org/10.57760/sciencedb.08307]. All publicly available datasets includes KMplot database, UCSC Genome Browser, ENCODE, and STRING database.

Research involving human participants, their data, or biological material

Policy information about studies with human participants or human data. See also policy information about sex, gender (identity/presentation), and sexual orientation and race, ethnicity and racism.

Reporting on sex and gender	No human participants
Reporting on race, ethnicity, or other socially relevant groupings	No human participants
Population characteristics	No human participants
Recruitment	No human participants
Ethics oversight	No human participants

Note that full information on the approval of the study protocol must also be provided in the manuscript.

Field-specific reporting

Please select the one below that is the best fit for your research. If you are not sure, read the appropriate sections before making your selection.

- [x] Life sciences
- [ ] Behavioural & social sciences
- [ ] Ecological, evolutionary & environmental sciences

For a reference copy of the document with all sections, see nature.com/documents/nr-reporting-summary-flat.pdf

Life sciences study design

All studies must disclose on these points even when the disclosure is negative.

Sample size	No statistical methods were employed to predetermine the sample sizes. All laboratory experiments were conducted with sample sizes that were deemed sufficient for the specific purposes of each experiment. In each group, a minimum of three samples were selected.
Data exclusions	No data were excluded from the analyses.
Replication	Information regarding experimental replication can be found in the Methods section and figure legends. All experiments were conducted with a minimum of three replicates, which was deemed necessary to ensure reliable and robust results.
Randomization	The samples were randomly assigned to the experimental groups .
Blinding	Blinding was not applied in this study. In the experimental phase, species names were distinguishable based on different cell morphology. In the bioinformatics analysis, the selection of the reference genome was based on the different species being studied.

Reporting for specific materials, systems and methods

We require information from authors about some types of materials, experimental systems and methods used in many studies. Here, indicate whether each material, system or method listed is relevant to your study. If you are not sure if a list item applies to your research, read the appropriate section before selecting a response.

Materials & experimental systems

n/a	Involved in the study	
☐	☒	Antibodies
☐	☒	Eukaryotic cell lines
☒	☐	Palaeontology and archaeology
☐	☒	Animals and other organisms
☒	☐	Clinical data
☒	☐	Dual use research of concern
☒	☐	Plants

Methods

n/a	Involved in the study	
☒	☐	ChIP-seq
☒	☐	Flow cytometry
☒	☐	MRI-based neuroimaging

Antibodies

Antibodies used	The protein levels of different genes were detected using the following primary antibodies: anti-HRAS(G12V) (1:500, CST, Rabbit, Cat. no. 14412S), anti- SV40 T Ag (1:500, Sant Cruz, Mouse, Cat. no. sc-147), anti-HIF1A(1:500, ZENBIO, Rabbit, Cat. no. 340462), anti-COPS5(1:1000, CST, Rabbit, Cat. no. 6895S), anti-RPS3(1:500, ZENBIO, Rabbit, Cat. no. R381083), anti-EIF5B(1:500, Affinity Bioscience; Rabbit, Cat. no. DF4055) and anti-EP300 (1:500, ZENBIO, Rabbit, Cat. no. 347220). The anti-β-actin (1:5000, sigma, Mouse, Cat. no. A5316-100µl) was used as a control. For rabbit or mouse polyclonal and monoclonal antibodies, the secondary antibodies used were anti-Rabbit (1:3000, CST, Cat. no. 7074S) and anti-Mouse (1:5000, Thermo Fisher, Cat. no. 31430). In the chromatin immunoprecipitation (ChIP) assay, the antibody HIF1A (10µl, CST, Cat. no.36169S) and rabbit IgG (1µl, Beyotime, Cat. no.A7016) were used.
Validation	All antibodies used in this study were commercially purchased and were validated by the manufacturers. The information regarding each antibody, including species and application, was obtained based on the catalog number (Cat. no.) provided by the manufacturers.

Eukaryotic cell lines

Policy information about cell lines and Sex and Gender in Research

Cell line source(s)	Primary skin fibroblasts from eight mammalian species were obtained and preserved in the in the CAS Kunming Cell Bank in the Kunming Institute of Zoology, Chinese Academy of Sciences, including laboratory mouse (Mus musculus, famale), the big-footed bat (Myotis pilosus, male), the Szechwan myotis (Myotis altarium, famale), the least horseshoe bat (Rhinolophus pusillus, male), the greater horseshoe bat (Rhinolophus ferrumequinum, male), the Chinese rufous horseshoe bat (Rhinolophus sinicus, male), the great leaf-nosed bat (Hipposideros armiger, male), and the Leschenault's Rousette (Rousettus leschenaultii, not collected). The cell lines of HEK293T, NIH3T3, HK-2, PANC-1, and MCF7 were purchased from the CAS Kunming Cell Bank at the Kunming Institute of Zoology, Chinese Academy of Sciences.
Authentication	None of the cell lines used were authenticated
Mycoplasma contamination	All cell lines tested negative for mycoplasma contamination
Commonly misidentified lines (See ICLAC register)	None

Animals and other research organisms

Policy information about studies involving animals; ARRIVE guidelines recommended for reporting animal research, and Sex and Gender in Research

Laboratory animals	Five to six-week-old B-NDG (NOD-Prkdcscid IL2rgtm1/Bcgen) mice were purchased from Jiangsu Biocytogen Co., Ltd (Nantong, China). All laboratory animals were housed in a pathogen-free environment.
Wild animals	The bats used in this study were captured from caves located in the southwest region of China. The bat species included the big-footed bat (Myotis pilosus), the Szechwan myotis (Myotis altarium), the least horseshoe bat (Rhinolophus pusillus), the greater horseshoe bat (Rhinolophus ferrumequinum), the Chinese rufous horseshoe bat (Rhinolophus sinicus), the great leaf-nosed bat (Hipposideros armiger), and the Leschenault's Rousette (Rousettus leschenaultii). Unfortunately, the age of the captured bats was unknown. Bats were captured using mist nets, and then they were carefully placed into bags for transportation with car. Euthanasia for the bats was performed using inhaled isoflurane. Subsequently, the bats were sacrificed to generate primary fibroblasts from different tissues.
Reporting on sex	The effect of sex was not considered in this study.
Field-collected samples	After being captured and brought back to the lab, bats were immediately sacrificed in order to obtain their different tissues.
Ethics oversight	All animals procedures were approved by the Ethics Committee of the Kunming Institute of Zoology, CAS.

Note that full information on the approval of the study protocol must also be provided in the manuscript.
